# Machine Learning Applied to CT Radiomics Identifies Symptomatic Carotid Plaques

**DOI:** 10.2174/0115734056431511260121053248

**Published:** 2026-04-01

**Authors:** Meilan Zhang, Jing Li, Yan Wang, Yani Zhao, Wanting Xie, Hai Du

**Affiliations:** 1 Graduate School, Baotou Medical College, Baotou, China; 2 Department of Radiology, Ordos Central Hospital, Ordos, China; 3 Department of Stroke, Ordos Central Hospital, Ordos, China

**Keywords:** Carotid plaque, Computed tomography angiography (CTA), Machine learning, Radiomics, Stroke, HDL-C

## Abstract

**Introduction:**

This study presents the development and validation of a multimodal framework that incorporates CT-based radiomics, machine learning algorithms, and clinico-radiologic features to enhance the detection accuracy of symptomatic carotid plaques. Clinically applicable nomograms were established for personalized risk stratification and management in asymptomatic patients.

**Methods:**

This retrospective study analyzed computed tomography angiography (CTA) data from 206 head and neck patients at Ordos Central Hospital (January 2020-June 2024), including 157 symptomatic and 49 asymptomatic individuals. Patients were randomly allocated into training/validation cohorts (7:3 ratio), with an additional prospectively enrolled cohort of 44 carotid plaque cases from Jining First People’s Hospital serving as an independent test set. Following radiomics feature extraction from CTA images, machine learning models were constructed using tree-based algorithms (Random Forest, XGBoost, LightGBM). An integrated multimodal model combining clinical variables, radiological characteristics, and radiomics signatures was developed. Performance was assessed *via* receiver operating characteristic (ROC) analysis, reporting area under the curve (AUC), sensitivity, and specificity metrics.

**Results:**

Multivariable analysis identified high-density lipoprotein cholesterol (HDL-C) levels and a history of diabetes as independent predictors of symptomatic carotid plaques. Radiomics models utilizing machine learning demonstrated moderate to strong diagnostic capability, yielding AUCs of 0.625-0.814 in the validation cohort and 0.743-0.802 in the test cohort. The multimodal integrated framework consistently surpassed standalone models, attaining AUCs of 0.836 in the validation set and 0.845 in the test set, which were significantly higher than both clinical and radiomics predictors alone.

**Discussion:**

The CT radiomics-based machine learning model developed in this study demonstrated favorable diagnostic efficacy in discriminating symptomatic carotid plaques. The multimodal framework integrating clinical indicators with radiomic signatures significantly enhanced the model's discriminative power and generalizability. This work confirms the clinical potential of radiomics analysis derived from routine CTA for precision risk assessment in cardiovascular disease, providing a novel supportive tool for individualized risk stratification of carotid atherosclerosis.

**Conclusion:**

CT imaging-based radiomics and machine learning models can effectively support clinical decision-making, enhance risk stratification for carotid plaque patients, and facilitate personalized treatment strategies.

## 1 INTRODUCTION

Globally, stroke constitutes the second leading cause of mortality [1]. Ischemic stroke, the most prevalent subtype, accounts for 69.6% to 70.8% of stroke cases in China [2, 3]. By 2020, approximately 20% of individuals aged 30 to 79 years worldwide were estimated to have carotid plaque [4]. Historically, the prediction of stroke risk predominantly depends on evaluating the extent of luminal stenosis resulting from plaques. A robust body of research now establishes plaque structural integrity and biochemical composition as critical mediators of thromboembolic potential, superseding traditional stenosis-based risk stratification [5]. Patients with high-risk plaque characteristics have been shown to have a threefold higher incidence of ipsilateral neurological ischemic events than those with stable plaques [6]. Therefore, the noninvasive detection of symptomatic carotid plaques may improve risk stratification, paving the way for more effective clinical management approaches.

Currently, carotid plaque evaluation in clinical practice primarily relies on CTA, a noninvasive imaging modality valued for its operational simplicity and high spatial resolution [7]. CTA enables the assessment of multiple plaque characteristics, including calcification, morphology, and stenosis severity. Diagnostic accuracy remains suboptimal for key high-risk plaque characteristics, including intraplaque hemorrhage (IPH) and lipid-rich necrotic core (LRNC) [8]. To overcome these constraints, radiomics has emerged as a novel approach that employs automated quantification of medical imaging biomarkers [9]. Radiomics has demonstrated broad applicability in disease diagnosis, prognosis prediction, and tumor classification/staging [10-12], with recent studies highlighting its role in cardiovascular plaque characterization [13-15]. With the growing emphasis on precision medicine [16], there is increasing recognition that diseases, particularly those with inherent heterogeneity, should be redefined based on underlying pathophysiological mechanisms, thereby uncovering novel therapeutic targets [17]. Investigating the pathogenesis of multifactorial diseases remains challenging, thereby accelerating the adoption of machine learning, a subfield of artificial intelligence (AI), as an evolving solution. By leveraging radiomic features, machine learning models can autonomously discern intricate patterns within diverse datasets, offering clinicians data-driven insights to enhance decision-making [18].

This research established and tested CT radiomics-driven machine learning algorithms to assess their efficacy in discriminating symptomatic carotid plaques. Improved symptomatic plaque identification enables personalized risk stratification and refined clinical decision-making for high-risk asymptomatic carotid plaques.

## 2 MATERIALS AND METHODS

### 2.1 Patient Population, Ethics Approval, and Definitions

**Fig. (1):**
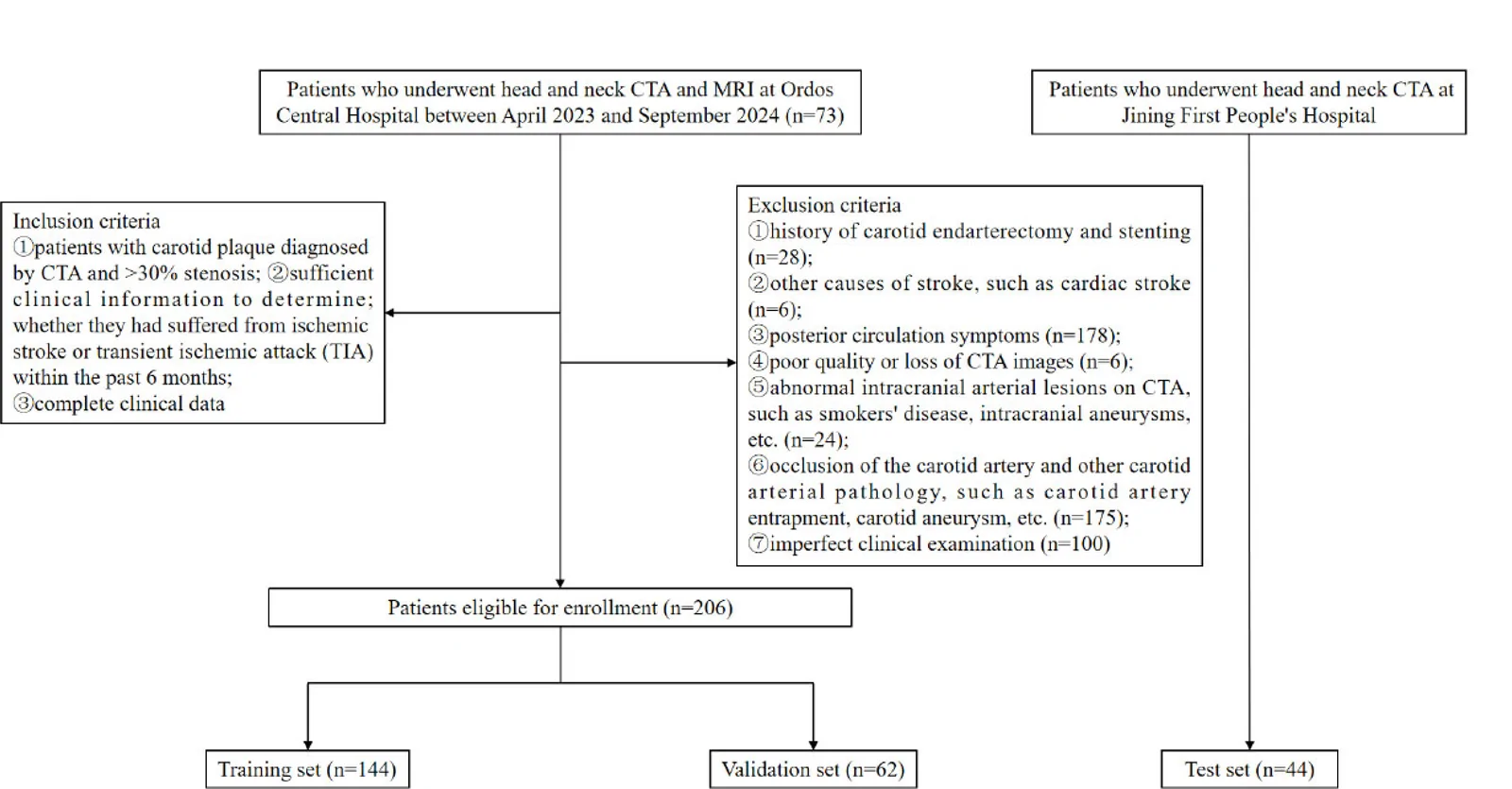
Flowchart: Patient exclusion criteria for symptomatic carotid plaque assessment. Note: n = number of patients.

This retrospective analysis was approved by the Ethics Committee of Ordos Central Hospital (Approval No. 2025-078) and was conducted in accordance with the ethical standards of the Declaration of Helsinki. This retrospective study consecutively enrolled head and neck CTA patients from Ordos Central Hospital between January 2020 and June 2024, with informed consent waived. Patient selection followed stringent inclusion/exclusion criteria as detailed in Fig. (**1**). The final cohort comprised 206 patients (157 symptomatic *vs.* 49 asymptomatic), randomly allocated to a training set and an internal validation set at a 7:3 ratio. An external test set of 44 patients from Jining First People's Hospital was enrolled for model generalizability validation, following identical exclusion criteria (Fig. **1**).

The sample size was determined by the availability of eligible patients during the study period, resulting in a cohort of 206 patients. This is consistent with the scale of similar retrospective radiomics studies [19]. Although larger sample sizes have been recommended [20], our model's robustness is strengthened by a prospectively enrolled external validation cohort (n=44), which mitigates overfitting concerns and supports generalizability.

Symptomatic cases comprised patients with ipsilateral ischemic events within ≤6 months: ocular (transient monocular blindness/blackout/retinal infarction) or cerebral hemispheric (TIA/stroke) [21]. Asymptomatic patients were defined as those without cerebrovascular symptoms. Systematically collected clinical data included age, sex, smoking history, and physician-diagnosed comorbidities, including diabetes mellitus (DM), hypertension (HTN), and coronary artery disease (CAD), medication use (antihypertensives, lipid-lowering drugs, antidiabetic agents, antiplatelet therapies), and fasting lipid profiles (total cholesterol [TC], triglycerides [TG], low-density lipoprotein cholesterol [LDL-C], and HDL-C). The study followed the Sex and Gender Equity in Re search guidelines (SAGER) to ensure appropriate inclusion and reporting of sex-related data.

### 2.2 Image Acquisition

A GE Revolution CT 256-slice scanner was used to scan patients in the supine position from the aortic arch to the cranial vault. After verifying venous patency *via* a 20 mL saline test bolus through the median cubital vein, 40-50 mL iohexol was injected (4.0-5.0 mL/s), followed by a 20 mL saline chase. Automated triggering occurred 2 seconds post-descending aortic attenuation reaching 245 HU. Images were analyzed on a GE AW 4.7 workstation.

The key scanning parameters included tube voltage: 100 kV; tube current: 100-350 mA (auto-modulation); matrix: 512 × 512; detector coverage: 40 mm; SFOV: 25.0 cm; reconstruction: 0.625 mm slice thickness and spacing.

### 2.3 Conventional CTA-derived Carotid Plaque Analysis

CTA images were analyzed on a post-processing workstation for carotid plaques located in the common carotid artery, carotid bifurcation (±2 cm), and extracranial internal carotid artery. The atherosclerotic plaque at the narrowest point was studied, with measurements of its maximum thickness, attenuation value, surface morphology, calcification presence, and stenosis severity.

The maximum thickness represents the maximal perpendicular diameter measured along the vascular axis [22]. Plaque CT value was measured by drawing ROIs from the lower density of the plaque, with an area of ROIs of at least 1 mm^2^ [23]. Using the same method, we measured the chosen section along with the two immediately adjacent sections and used the average of these three values as the final CT attenuation. Calcification was defined as the portion of the plaque with a CT value greater than 130 HU [24]. Plaque volume was automatically calculated by the software following manual outlining of the plaque using ITK-SNAP (version 3.8.0, www.itksnap.org), as illustrated in Fig. (**2**).

Carotid artery stenosis was quantified as the percentage reduction in luminal diameter according to the North American Symptomatic Carotid Endarterectomy Trial (NASCET) criteria [25], using the following formula:

Stenosis (%) = [1 − (Minimal residual lumen diameter / Normal distal lumen diameter)] × 100%. Stenosis severity was categorized as mild (<30%), moderate (30%–69%), or severe (70%–99%).

Plaque surface morphology was defined as ulcerated if there was at least 1 mm of contrast entry within the plaque, and if there was contrast enhancement within the plaque of between 0.3 mm and 0.9 mm, the surface was defined as irregular; otherwise, the surface was defined as smooth. Qualitative criteria were assessed by two radiologists with five years of experience in cardiovascular imaging. For controversial imaging findings, consultation was conducted with a senior radiologist possessing over 20 years of specialized experience until consensus was achieved.

All collected features underwent univariate and multivariate screening, with variables demonstrating statistical significance (*p* < 0.05) retained for clinical model development.

### 2.4 Image Segmentation

**Fig. (2):**
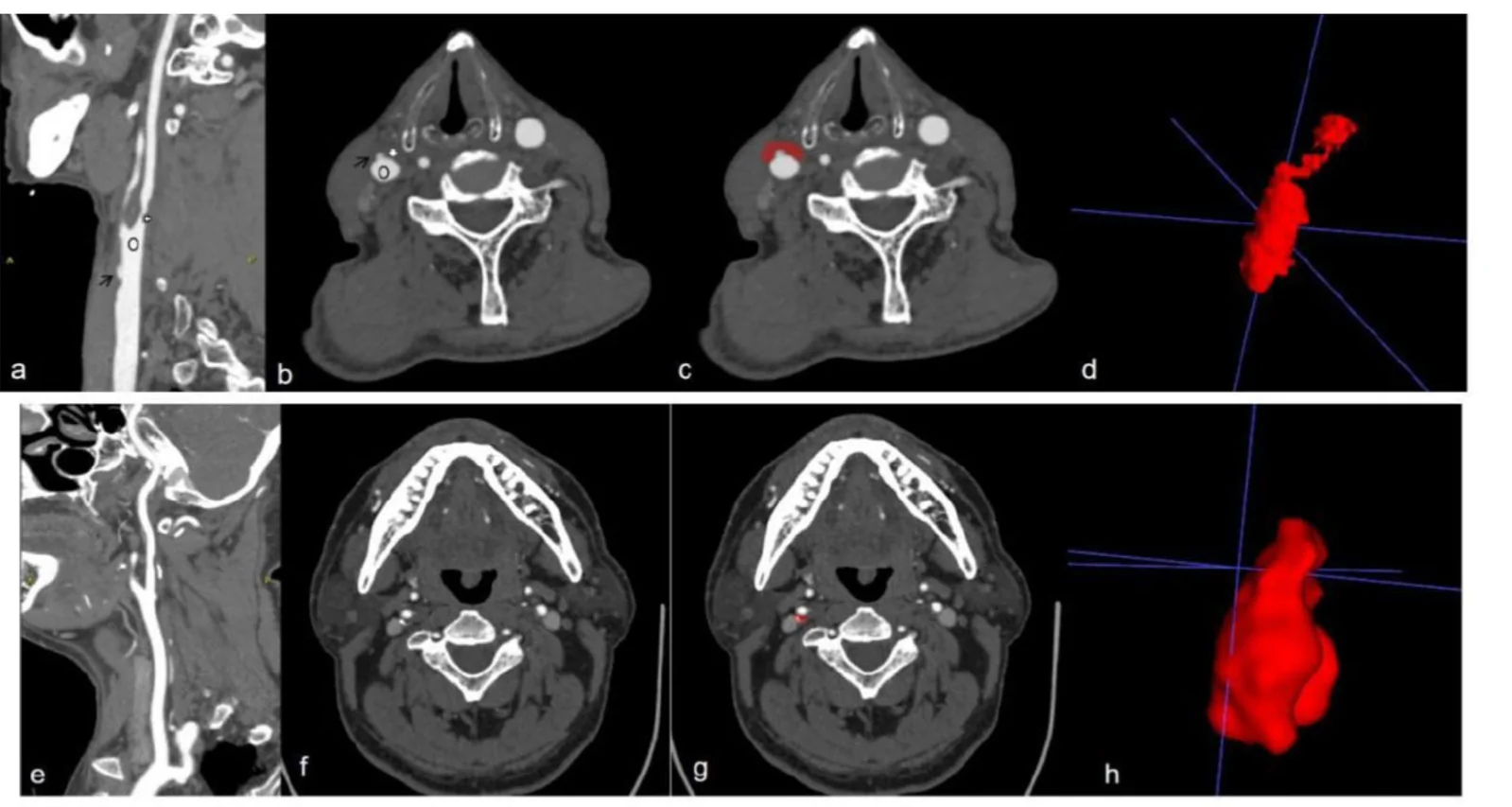
Representative examples of symptomatic vs. asymptomatic carotid plaques and corresponding volume-of-interest (VOI) acquisition. **(a–d)** Symptomatic plaque in a 74-year-old male at the right carotid bifurcation, showing severe stenosis with ulceration (black arrow) at the point of maximal plaque thickness. The white cross marks the plaque centroid, and the black circle indicates the residual lumen. (**c**) shows the region of interest (ROI) delineation. (**d**) illustrates the 3D VOI reconstruction. **(e–h)** Asymptomatic plaque in a 57-year-old male at the right internal carotid artery origin, demonstrating moderate stenosis without high-risk features. (**g**) displays the ROI segmentation. (**h**) presents the VOI extraction.

Two board-certified radiologists (5 years' experience) segmented plaques layer-wise using ITK-SNAP to generate 3D volumes of interest (VOIs). These VOIs encompassed both calcified and non-calcified plaque components. The outlined VOIs were stored in NIfTI (.nii) format (Fig. **2**). Consultation with senior radiologists with more than 20 years of professional experience for any disagreeable parts ensured a high degree of annotation accuracy and reliability. Intraclass correlation coefficients were also calculated.

### 2.5 Radiomics Feature Extraction

Images were collected in DICOM format, resampled to an isotropic 1×1×1 mm^3^ voxel size, intensity-normalized, and applied to the Laplacian of Gaussian and Wavelet filters. Feature extraction was performed across the entire Volume of Interest (VOI) to ensure comprehensive plaque representation. In clustered subregions with unclassified areas, the K-Nearest Neighbors (KNN) algorithm was used to assign consistent labels. Radiomic feature extraction was performed using PyRadiomics (v3.0.1; https://github.com/Radiomics/pyradiomics), an open-source Python library for medical image analysis, in accordance with the Imaging Biomarker Standardization Initiative (IBSI) guidelines to ensure methodological consistency and reproducibility. Features underwent Z-score normalization (mean=0, SD=1) for standardization. Univariate analysis retained features with *p*-values of <0.05. Redundant features were eliminated by removing those with Pearson correlation coefficients >0.9.

To refine the radiomic feature set and reduce redundancy, a multi-step feature selection pipeline was applied.

Statistical Filtering: Features with *p* < 0.05 in univariate analysis were retained.

Correlation Analysis: Features exhibiting high collinearity (Pearson’s correlation coefficient ≥ 0.9) were excluded.

LASSO Regression: Least Absolute Shrinkage and Selection Operator (LASSO) regression was employed to identify the most predictive features. The optimal regularization parameter (λ) was determined through 10-fold cross-validation, performed once without repetition, using sequentially partitioned data without shuffling. To ensure reproducibility within this specific experimental setup, all analyses were conducted within consistent software environments on a fixed dataset. It should be noted that explicit randomization controls (*e.g.*, setting random seeds) were not applied during this process.

### 2.6 Model Development and Evaluation

The clinical model was constructed using clinical variables selected *via* univariate and multivariate analyses (*p* < 0.05).

Filtered radiomic features were utilized to construct machine learning models *via* three tree-based algorithms (Random Forest [RF], XGBoost, and LightGBM) and one fusion-based variant of LightGBM.

The combined model integrated both clinically significant features identified through statistical analysis and radiomic predictors.

Its performance was comprehensively evaluated using ROC curve analysis (assessing AUC, sensitivity, and specificity), nomogram visualization, and decision curve analysis (DCA) to quantify net clinical benefit across various probability thresholds.

### 2.7 Statistical Analysis

Statistical analyses were performed using SPSS 26.0 and Python 3.7.12. Categorical data were expressed as frequencies (%) with chi-square comparisons and continuous variables as mean±SD. After normality assessment, normally distributed variables were analyzed with independent t-tests, while non-normally distributed variables were analyzed with Mann-Whitney U tests. A two-tailed p-value <0.05 was defined as statistical significance. Interobserver agreement was quantified using the intraclass correlation coefficient (ICC). An ICC value below 0.5 was interpreted as poor agreement, 0.5–0.75 as moderate, 0.75–0.9 as good, and above 0.9 as excellent.

An a priori sample size calculation was conducted to ensure adequate statistical power. Using the MedSci Sample Size Tools (MSST, version 6.4.9), which implements the established methodology for diagnostic studies by Obuchowski and Li [26, 27], we determined that a minimum of 154 participants was required. This was based on an expected sensitivity of 0.80, a specificity of 0.85, a margin of error of 0.05 for both indices, and a two-sided α of 0.05. After accounting for an estimated 10% drop-out rate, the target sample size was 171. Our final cohort of 206 patients satisfied this requirement. Furthermore, this sample size was consistent with those reported in similar high-impact studies utilizing radiomics or advanced imaging for carotid plaque characterization, which typically enrolled between 100 and 250 patients [28, 29].

To construct the multivariate logistic regression model, candidate variables with a p-value < 0.05 from the univariate analysis were initially included to identify independent predictors. This less stringent threshold helps retain potential confounding factors that might not reach traditional significance but could still be clinically relevant. The final multivariate model was then refined using a stepwise backward elimination procedure with a significance level of *p* < 0.05 for retention. This process iteratively removes variables that do not contribute significantly to the model when adjusted for other factors.

## 3 RESULTS

### 3.1 Analysis of Clinical Signs

Table **1** summarizes clinical characteristics. Significant predictors of symptomatic carotid plaques identified through univariate and multivariate analyses are presented in Table **2**. Univariate analysis revealed associations between symptomatic plaques and HDL-C, diabetes history, and plaque location (*p*<0.05). Multivariate analysis revealed HDL-C (OR=0.783; 95%CI:0.678-0.904) and diabetes history (OR=0.848; 95%CI:0.770-0.933) as significant independent protective factors. Plaque location demonstrated persistent protective effects (OR=0.92; 95%CI:0.871-0.972).

### 3.2 Comparison of Imageomics Model Construction and Effectiveness

**Fig. (3):**
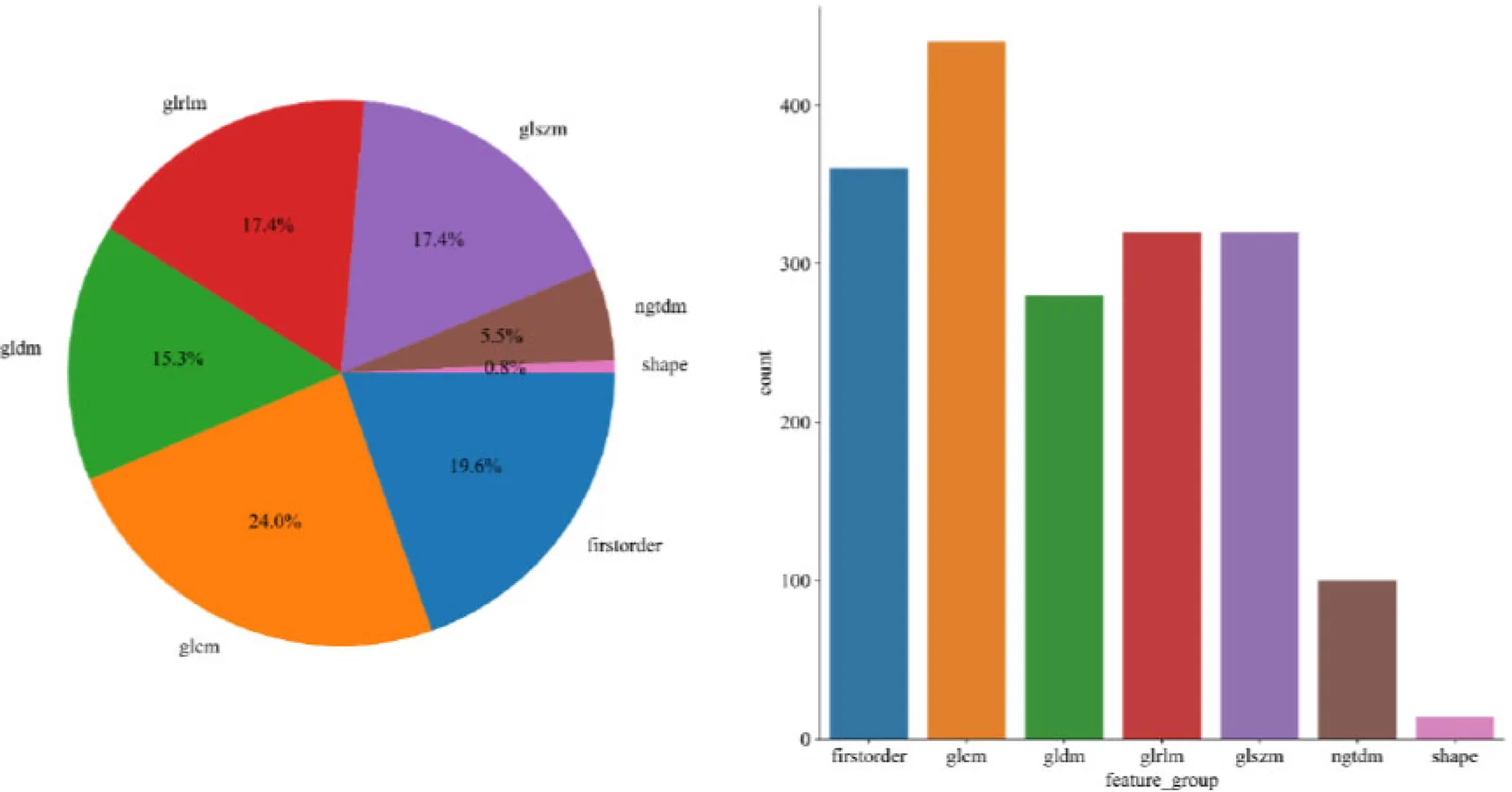
Number and percentage of imaging histology features.

Radiomic analysis of CTA images extracted 1,834 features (Fig. **3**), comprising 14 shape-based, 360 first-order statistical, and 1,460 textural descriptors. The shape features mainly described the geometric morphology and volume distribution of the plaque, the first-order statistics reflected the gray-scale distribution within the plaque, and the texture features contributed more to the model, especially those based on the Gray-Level Co-occurrence Matrix (GLCM), which were effective in capturing the heterogeneity within the plaques, and thus helped to distinguish between symptomatic and asymptomatic plaques. Moreover, a 10-fold cross-validated LASSO regression selected key predictors.

**Fig. (4):**
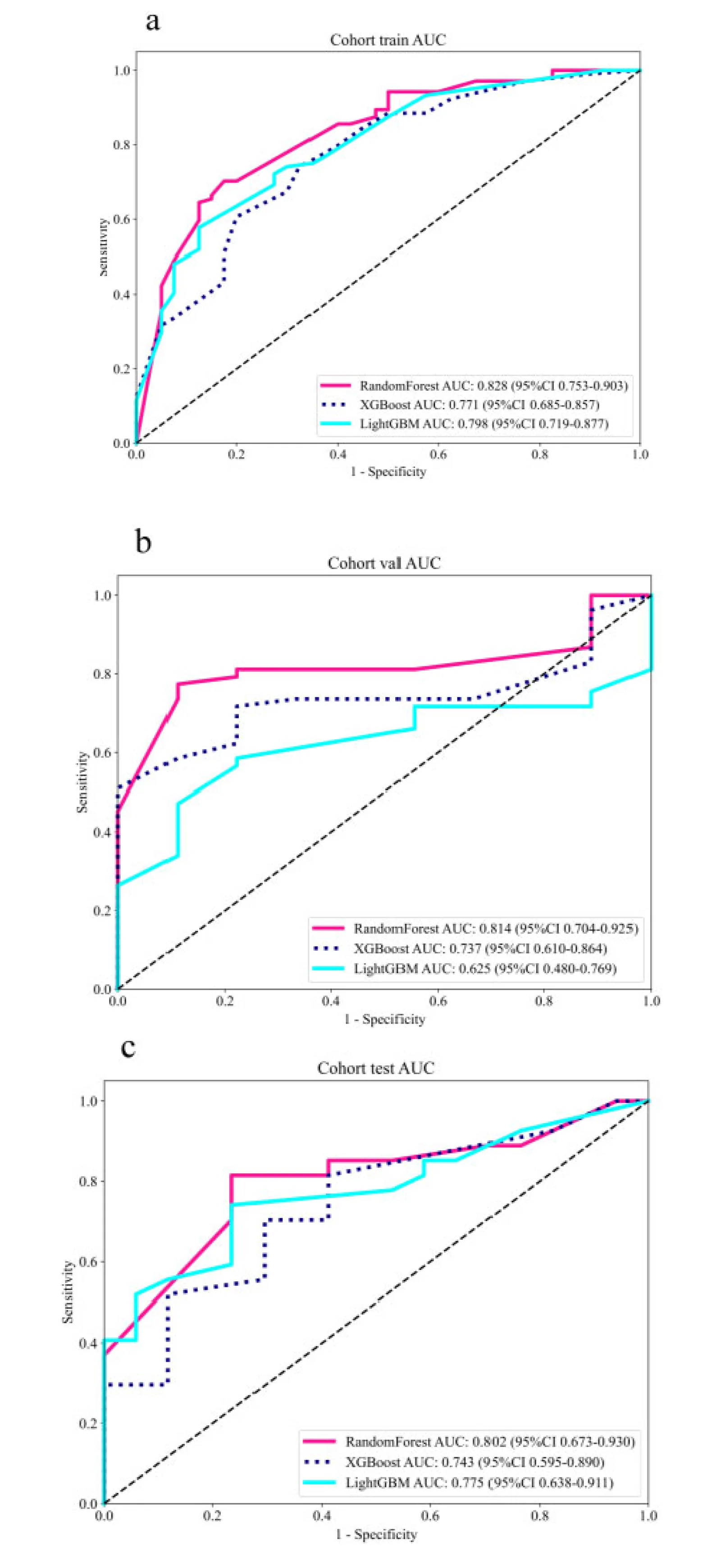
ROC curves of radiomics-based ML models across training/validation/test sets (**a**: TR, **b**: VL, **c**: TS).

Radiomic models achieved AUCs ranging from 0.625 to 0.814 across validation/test cohorts (Table **3**). Although statistical comparison using DeLong's test revealed no significant difference in AUC between the combined model and the best-performing radiomics model (*p* > 0.05), the diagnostic abilities of the three machine learning models were notably consistent (Fig. **4**). This consistency indicates a robust ability and stability of the imaging histology features themselves in discriminating symptomatic carotid plaques.

### 3.3 Combined Model Construction and Efficacy Comparison

**Fig. (5):**
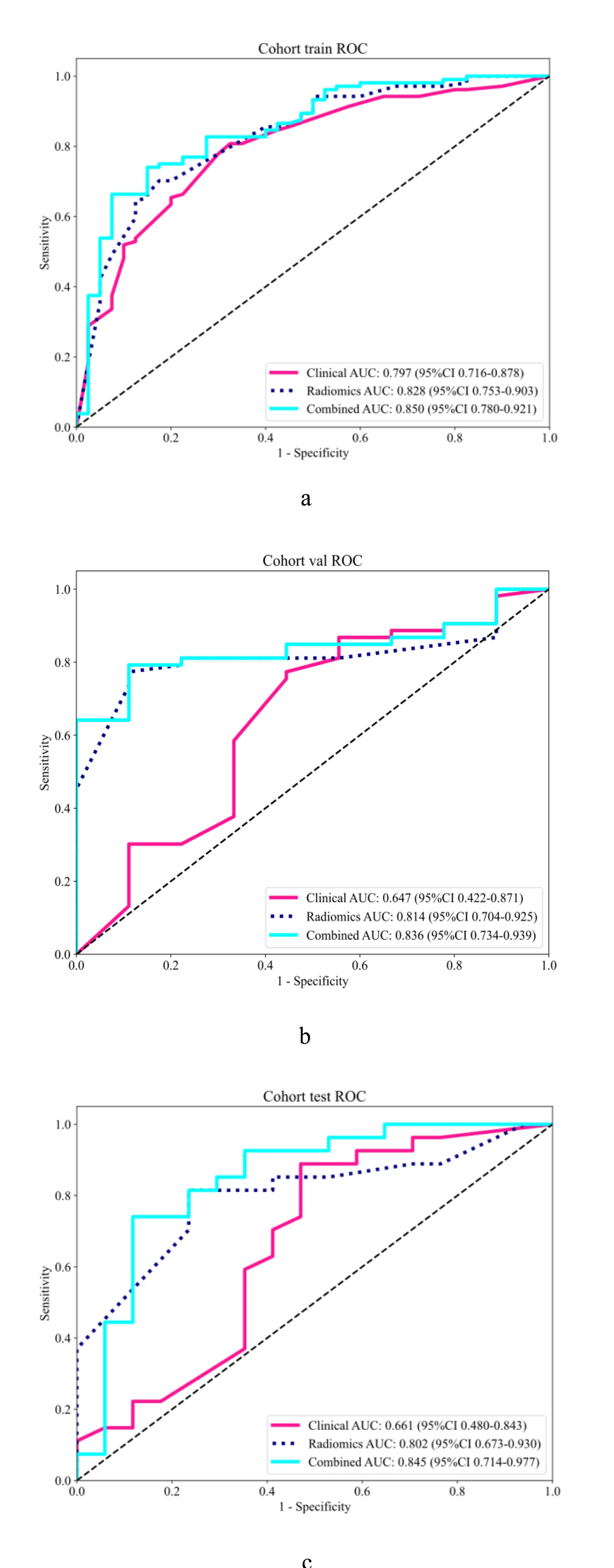
ROC analysis of clinical, radiomic, and integrated models across datasets (**a**: Train, **b**: Valid, **c**: Test).

**Fig. (6):**
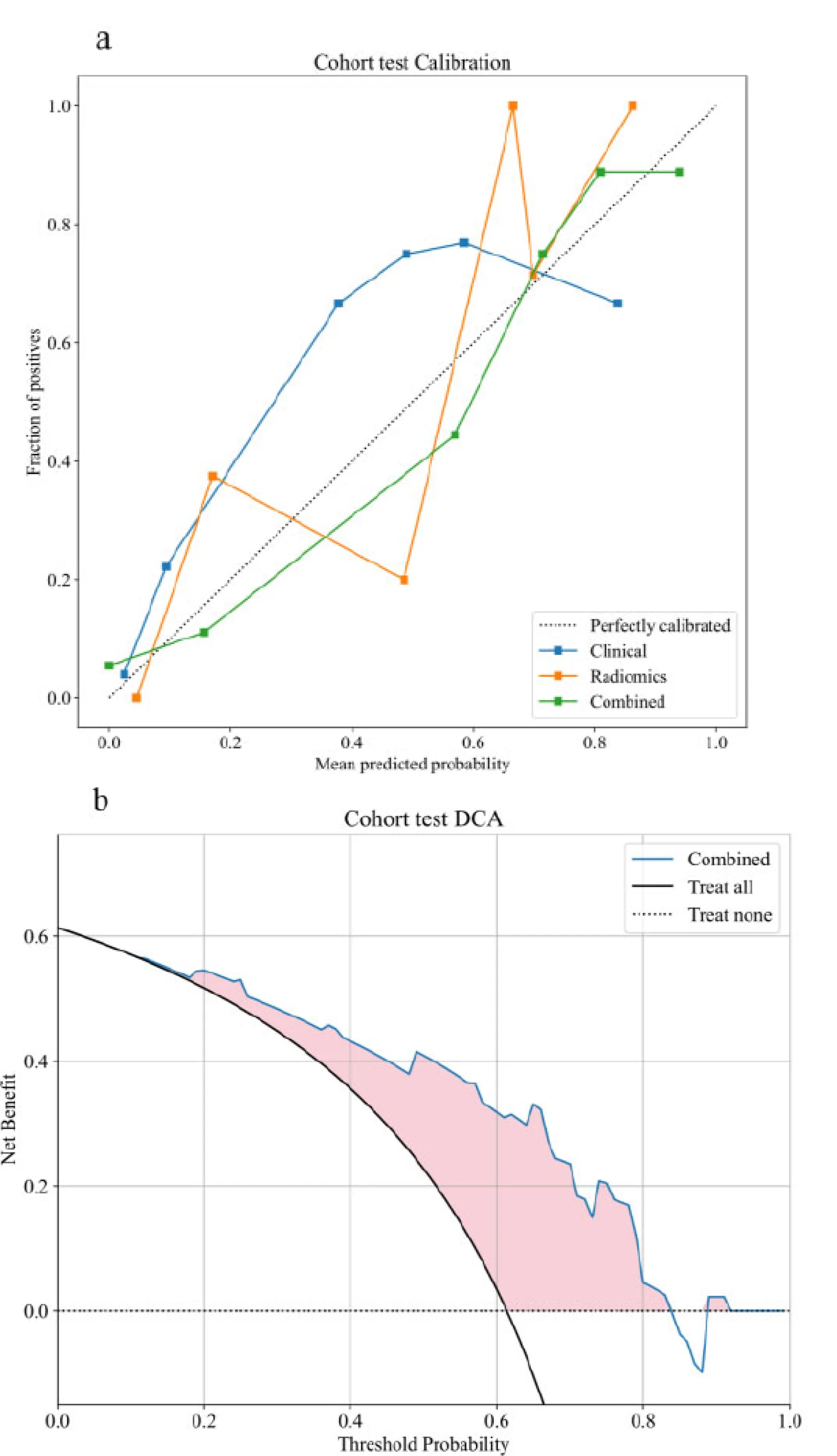
RF model calibration curves (**a**) and decision analysis curves (**b**) in the test set.

Among the three machine learning models, Random Forest performed best across all datasets. The combined model integrated clinical and radiomic features using Random Forest (Fig. **5**), achieving significantly higher AUCs than the individual models: 0.836 [0.733–0.9^39^] in the validation set and 0.845 [0.741–0.977] in the test set (Table **4**). This combined approach allows for a more comprehensive assessment of symptomatic carotid plaques by capturing multi-dimensional features. For instance, clinical features, such as HDL-C and diabetes history, offer insights into the patient's metabolic status, while imaging histology features provide detailed information on plaque morphology and texture, thereby offering a more robust foundation for clinical decision-making. In the test set, visual inspection of calibration curves confirmed precise alignment of logistic regression (LR) predictions with observed symptomatic plaque prevalence (Fig. **6a**). Although quantitative metrics, such as the Brier score, were not computed, the visual closeness of the calibration curve to the ideal diagonal line suggests acceptable calibration performance. Similarly, decision curve analysis (Fig. **6b**) visually demonstrated that the integrated model provided a superior net benefit across a wide range of clinically reasonable probability thresholds compared to the 'treat-all' or 'treat-none' strategies.

### 3.4 Nomogram Construction

**Fig. (7):**
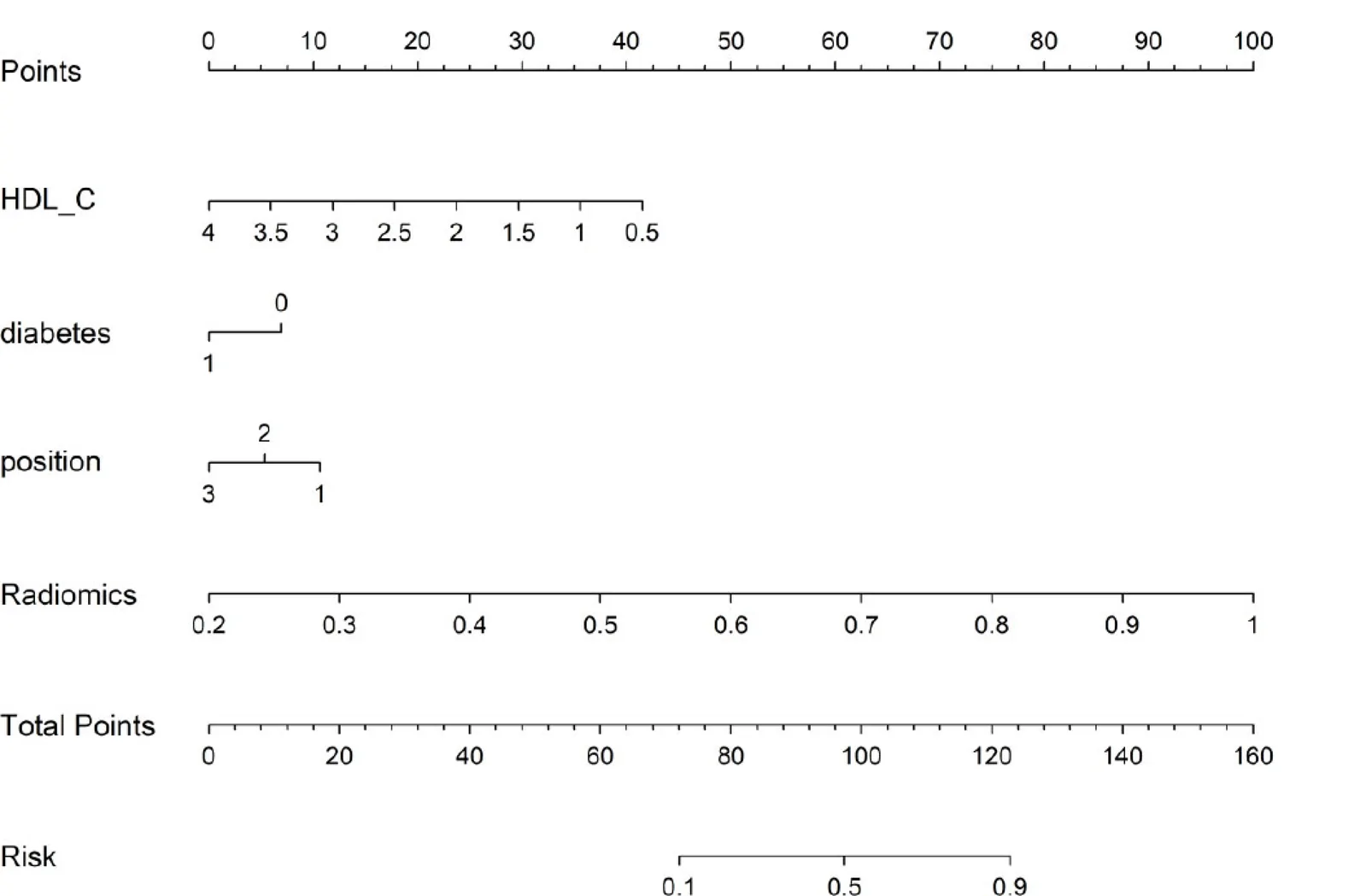
Nomogram construction.

To facilitate clinical application, this study developed a nomogram (Fig. **7**) based on the combined model. This tool visually illustrates each feature's contribution to the risk of symptomatic carotid plaque, enabling clinicians to rapidly assess risk based on the patient's specific features. This provides a basis for personalized treatment strategies.

## 4 DISCUSSION

Machine learning models based on CT imaging histological features demonstrate promising diagnostic efficacy for identifying symptomatic carotid plaques. These models may inform clinical decision-making and personalized management approaches, potentially enhancing risk stratification for patients with carotid plaques, pending further validation across diverse clinical settings. However, further research is needed to optimize the models and validate their stability and reliability across larger, multicenter datasets.

### 4.1 Clinical Significance of Differentiating Symptomatic *vs*. Asymptomatic Carotid Plaques

With the advances in pharmacological treatment of atherosclerosis, the European Society for Vascular Surgery has recommended that the treatment strategies for symptomatic *versus* asymptomatic carotid plaques should be different [30], with symptomatic carotid plaques mostly requiring clinical interventions, such as surgery or stenting, whereas asymptompto-matic carotid plaques are more often tmatic carotid plaques are more often treated with pharmacological treatments, with the goal of stabilizing the plaque. Differentiating symptomatic from asymptomatic carotid plaques enables tailored clinical management. CT radiomics-based machine learning models demonstrated robust discriminative capacity for carotid plaque classification, with integrated models achieving AUCs of 0.836 (validation) and 0.845 (test). This aligns with the findings of a study by Zaccagna *et al.* (AUC: 0.81) [31], confirming that imaging histology provides a non-invasive approach to quantify plaque vulnerability, where compositional features (*e.g.*, lipid-rich necrotic core, intraplaque hemorrhage) better predict rupture risk than luminal stenosis alone.

### 4.2 Advantages of Combining Clinical and Radiomic Features

In this study, reduced HDL-C levels and a history of diabetes emerged as independent protective factors against symptomatic carotid plaques. Low HDL-C is known to promote macrophage-driven plaque destabilization, thereby increasing rupture risk due to its weakened anti-inflammatory and antioxidant properties [32]. In contrast, poor glycemic control in diabetic patients may lead to impaired vascular endothelial function, further exacerbating plaque instability [33].

Carotid plaque vulnerability is influenced not only by luminal stenosis but also by pathophysiological features, such as vascular inflammation, intraplaque hemorrhage, and fibrous cap integrity [34, 35]. Plaque components exhibit distinct CT attenuation characteristics, yet partial volume effects hinder visual discrimination of intraplaque features. The clinical significance of radiomics lies in its ability to decode subclinical information for precision medicine, as demonstrated by its application in predicting ischemic stroke risk from intraplaque characteristics [31, 36, 37]. Our study directly contributes to this field by developing a radiomics model that specifically addresses the unmet clinical need for reliable, non-invasive risk stratification of symptomatic carotid plaques. In patients with >50% carotid stenosis, radiomics signatures significantly outperformed conventional features for symptomatic plaque detection (AUC: 0.858 *vs.* 0.706; *P*<0.01) [38]. The present study included carotid plaques with stenosis >30%, which had a wider range of interpretation for symptomatic plaques. Shi J. *et al.* [39] also found that that the imaging histology model had better diagnostic efficacy for symptomatic carotid plaques (AUC 0.767, 0.797, respectively) compared to the traditional model constructed by incorporating only clinical information (homocysteine) and imaging features (including plaque ulceration and carotid rim sign), in addition to the fact that multiple wavelet transform features were extracted [39], which is consistent with the present study. In our analysis, the screened imaging histology feature, intra_wave-let_HLH_firstorder_Mean, is one of the wavelet transform features, which can decompose the image into subbands of different frequencies and orientations to capture the local texture and structural information in the image. For symptomatic carotid plaques, the internal structure tends to be more complex, and these components lead to changes in gray value; therefore, this feature can be an important indicator for distinguishing symptomatic from asymptomatic plaques. Radiomics signatures derived from CT features achieved a test-set AUC of 0.786, confirming clinically applicable discriminative capacity for symptomatic carotid plaques.

### 4.3 Technical Validation and Clinical Applicability

Beyond its technical performance, the primary clinical value of our combined model lies in its incremental utility over conventional CTA reading, which relies on subjective morphological descriptors that may overlook textural heterogeneity indicative of plaque vulnerability. By providing an objective “vulnerability score” derived from subvisual patterns, our model enhanced risk stratification, particularly in patients with moderate stenosis (30%–69%), where management decisions are most ambiguous. To ensure clinical applicability, we deliberately employed shallow learning classifiers (RF, XGBoost, LightGBM) [40] for three key reasons. First, the limited dataset size favored shallow algorithms' robustness over deep learning, which typically requires larger datasets to avoid overfitting [41]. Second, and crucially, tree-based models offer intrinsic interpretability through feature importance scores, which is paramount for building clinical trust. As highlighted by *Abrames* and *Rouzrokh*, while complex models like deep neural networks are often explainable through post-hoc techniques, intrinsically interpretable models are easier for humans to understand directly, fostering greater adoption among clinicians [42]. Finally, the computational efficiency of shallow learning facilitates integration into routine workflows without specialized hardware. The RF-based model achieved validation/test AUCs of 0.836/0.845, consistent with the radiomics study by Dong *et al.* (AUC = 0.858) [38], but surpassing its single-center design through enhanced generalizability (n = 707 *vs.* 120). This demonstrates that for well-defined clinical imaging tasks, sophisticated shallow learning can be the most practical and effective path to a clinically applicable solution. The model can be embedded as a PACS plugin to automatically analyze plaques and augment radiological reports, enabling early high-risk patient identification while reducing unnecessary procedures, all without additional scanning costs.

### 4.4 Limitations

This study has several limitations, which are as follows:

(1) Retrospective design with potential selection bias.

(2) The generalizability of our findings is constrained by the retrospective, single-center design of the primary cohort. Furthermore, while the inclusion of an external validation cohort from a different institution is a strength, its relatively small sample size (n=44) may limit the statistical power and stability of the performance estimates for robust testing. Therefore, our results, particularly the model's performance on the external test set, should be interpreted as preliminary evidence of generalizability and require confirmation in larger, prospective, multi-center studies.

(3) Inability to fully exclude competing etiologies for ischemic events despite rigorous exclusion criteria.

(4) Quantitative calibration metrics (*e.g.*, Brier score) and decision curve analysis were not provided. Including these in future studies would allow more objective evaluation of model calibration and clinical utility.

(5) The present study may have been underpowered to detect a statistically significant difference in performance between the combined model and the best standalone models, despite the observed numerical improvement in AUC. This is likely attributable to the limited sample size, and the potential superiority of the integrated approach needs to be confirmed in larger studies.

## CONCLUSION

In summary, CT radiomics-based machine learning models show promising diagnostic performance for symptomatic carotid plaque detection. These tools may support clinical decisions and personalized treatment planning, providing non-invasive risk stratification. Further optimization and multicenter validation are required to establish clinical robustness.

## Figures and Tables

**Table 1:** Clinical characteristics of the study population.

**Feature_Name**	**Train**	**Val**	**Test**	***P*-value**
Age(years, 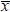 ±s)	68.62±8.37	67.00±7.81	71.48±6.80	0.02
TC(mmol/L, 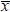 ±s)	4.31±1.07	3.97±1.21	4.06±1.31	0.111
TG(mmol/L, 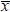 ±s)^a^	1.70±1.08	1.58±0.79	1.46±1.26	0.393
HDL_C(mmol/L, 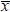 ±s)^a^	1.02±0.35	0.95±0.23	1.15±0.29	0.007
LDL_C(mmol/L, 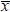 ±s)^a^	2.91±1.03	2.81±1.14	2.50±1.08	0.085
Maximum Plaque Thickness(mm, 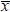 ±s)^a^	4.03±1.26	4.61±2.53	4.72±1.40	0.015
CT Value(HU, 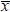 ±s)^a^	65.97±170.03	64.91±134.44	99.54±269.76	0.541
Plaque Volume(mm^3^, 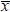 ±s)^a^	791.53±798.31	962.99±674.71	912.39±550.18	0.260
Gender, n(%)^b^	-	-	-	0.158
Female	35(24.31)	8(12.90)	11(25.00)	-
Male	109(75.69)	54(87.10)	33(75.00)	-
Position, n(%)^b^	-	-	-	0.083
left side	41(28.47)	16(25.81)	10(22.73)	-
Right side	42(29.17)	26(41.94)	9(20.45)	-
Bilateral	61(42.36)	20(32.26)	25(56.82)	-
The degree of stenosis, n(%)^b^	-	-	-	0.029
Mild	38(26.39)	14(22.58)	3(6.82)	-
Moderate	54(37.50)	25(40.32)	15(34.09)	-
Severe	52(36.11)	23(37.10)	26(59.09)	-
CHD, n(%)^b^	-	-	-	0.231
0	106(73.61)	50(80.65)	29(65.91)	-
1	38(26.39)	12(19.35)	15(34.09)	-
Hypertension, n(%)^b^	-	-	-	0.228
0	49(34.03)	21(33.87)	21(47.73)	-
1	95(65.97)	41(66.13)	23(52.27)	-
Diabetes, n(%)^b^	-	-	-	0.509
0	92(63.89)	39(62.90)	32(72.73)	-
1	52(36.11)	23(37.10)	12(27.27)	-
Smoking, n(%)^b^	-	-	-	0.798
0	84(58.33)	38(61.29)	28(63.64)	-
1	60(41.67)	24(38.71)	16(36.36)	-
Application of antiplatelet drugs, n(%)^b^	-	-	-	0.325
0	89(61.81)	39(62.90)	22(50.00)	-
1	55(38.19)	23(37.10)	22(50.00)	-
Application of antihyperglycemic drugs, n(%)^b^	-	-	-	0.193
0	94(65.28)	41(66.13)	35(79.55)	-
1	50(34.72)	21(33.87)	9(20.45)	-
Application of antilipidemic drugs, n(%)^b^	-	-	-	0.976
0	91(63.19)	39(62.90)	27(61.36)	-
1	53(36.81)	23(37.10)	17(38.64)	-
Application of antihypertensive drugs, n(%)^b^	-	-	-	0.024
0	54(37.50)	22(35.48)	26(59.09)	-
1	90(62.50)	40(64.52)	18(40.91)	-
Calcification, n(%)^b^	-	-	-	0.122
0	13(9.03)	5(8.06)	0	-
1	131(90.97)	57(91.94)	44(100.00)	-
Morphology of the plaque surface, n(%)^b^	-	-	-	0.363
Smooth	104(72.22)	40(64.52)	26(59.09)	-
irregular	22(15.28)	10(16.13)	11(25.00)	-
ulcerated	18(12.50)	12(19.35)	7(15.91)	-

**Note:** ^a^Continuous variables are expressed as mean ± standard deviation;^b^Categorical variables are expressed as frequency and percentage. **Note:** In this table, ‘1’ indicates the presence of the feature, and ‘0’ indicates its absence.

**Table 2:** Univariate and multivariate analysis of clinical characteristics.

**Feature_name**	**Univariate OR**	**95%CI**	***P*-value**	**MultivariateOR**	**95%CI**	***P*-value**
HDL_C	0.840	0.7280-0.9700	<0.05	0.783	0.6780-0.9040	<0.001
Calcification	0.848	0.7100-1.0130	0.128	-	-	-
Diabetes	0.868	0.7890-0.9550	<0.05	0.848	0.7700-0.9330	<0.001
Application of antihyperglycemic drug	0.914	0.8290-1.0090	0.134	-	-	-
Position	0.921	0.8710-0.9740	<0.05	0.92	0.8710-0.9720	<0.05
Application of antiplatelet drugs	0.942	0.8570-1.0350	0.294	-	-	-
Application of antilipidemic drugs	0.943	0.8570-1.0370	0.308	-	-	-
TC	0.956	0.9190-0.9950	0.062	-	-	-
CHD	0.962	0.8660-1.0690	0.549	-	-	-
LDL_C	0.977	0.9360-1.0200	0.369	-	-	-
TG	0.998	0.9550-1.0430	0.937	-	-	-
Plaque volume	1.000	1.0000-1.0000	0.74	-	-	-
CT value	1.000	1.0000-1.0000	0.123	-	-	-
Age	1.001	0.9950-1.0060	0.894	-	-	-
Maximum plaque thickness	1.021	0.9930-1.0480	0.216	-	-	-
The degree of stenosis	1.028	0.9690-1.0920	0.441	-	-	-
Morphology of the plaque surface	1.044	0.9810-1.1120	0.254	-	-	-
Application of antihypertensive drugs	1.052	0.9580-1.1560	0.372	-	-	-
Hypertension	1.053	0.9570-1.1580	0.377	-	-	-
Gender	1.067	0.9540-1.1940	0.341	-	-	-
Smoking	1.076	0.9790-1.1820	0.199	-	-	-

**Note:** OR, odds ratio; CI, confidence interval.

**Table 3:** Diagnostic performance of three machine learning models (Random Forest, XGBoost, and LightGBM) evaluated on training, validation, and test datasets.

	Accuracy	AUC	95% CI	Sensitivity	Specificity	PPV	NPV	Set
RandomForest	0.715	0.828	0.753 - 0.903	0.663	0.850	0.920	0.493	Train
RandomForest	0.774	0.814	0.704 - 0.925	0.755	0.889	0.976	0.381	Val
RandomForest	0.727	0.802	0.673 - 0.930	0.704	0.765	0.826	0.619	Test
XGBoost	0.681	0.771	0.685 - 0.857	0.673	0.700	0.854	0.452	Train
XGBoost	0.500	0.737	0.610 - 0.864	0.415	1.000	1.000	0.225	Val
XGBoost	0.682	0.743	0.595 - 0.890	0.667	0.706	0.783	0.571	Test
LightGBM	0.618	0.798	0.719 - 0.877	0.519	0.875	0.915	0.412	Train
LightGBM	0.597	0.625	0.480 - 0.769	0.566	0.778	0.937	0.233	Val
LightGBM	0.705	0.775	0.638 - 0.911	0.667	0.765	0.818	0.591	Test

**Table 4:** Diagnostic performance of clinical, radiomic, and integrated rf-derived models across training/validation/test cohorts.

	Accuracy	AUC	95% CI	Sensitivity	Specificity	PPV	NPV	Set
Clinical	0.757	0.797	0.7162 - 0.8775	0.779	0.700	0.871	0.549	Train
Radiomics	0.715	0.828	0.7531 - 0.9031	0.663	0.850	0.920	0.493	Train
Combined	0.764	0.850	0.7795 - 0.9212	0.731	0.850	0.927	0.548	Train
Clinical	0.597	0.647	0.4221 - 0.8714	0.585	0.667	0.912	0.214	Val
Radiomics	0.774	0.814	0.7041 - 0.9249	0.887	0.111	0.855	0.143	Val
Combined	0.806	0.836	0.7338 - 0.9392	0.792	0.889	0.977	0.421	Val
Clinical	0.500	0.661	0.4797 - 0.8428	0.407	0.647	0.647	0.407	Test
Radiomics	0.659	0.802	0.6731 - 0.9304	0.889	0.294	0.667	0.625	Test
Combined	0.795	0.845	0.7141 - 0.9765	0.926	0.588	0.781	0.833	Test

## Data Availability

All data generated or analyzed during this study are included in this published article.

## LIST OF ABBREVIATIONS

CTA: Computed tomography angiography
ROC: Receiver operating characteristic
AUC: Area under the curve
HDL-C: High-density lipoprotein cholesterol
IPH: Intraplaque hemorrhage
LRNC: Lipid-rich necrotic core
AI: Artificial intelligence
TIA: Transient ischemic attack
DM: Diabetes mellitus
HTN: Hypertension
CAD: Coronary artery disease
TC: Total cholesterol
TG: Triglycerides
LDL-C: Low-density lipoprotein cholesterol
HU: Hounsfield units
NASCET: North American Symptomatic Carotid Endarterectomy Trial
IBSI: Imaging Biomarker Standardization Initiative
LASSO: Least Absolute Shrinkage and Selection Operator
ICC: Intraclass correlation coefficient
DCA: Decision curve analysis
GLCM: Gray-level co-occurrence matrix
LR: Logistic regression

## STANDARDS OF REPORTING

STROBE guidelines were followed.
